# Nutritional Characteristics and Antimicrobial Activity of Australian Grown Feijoa (*Acca sellowiana*)

**DOI:** 10.3390/foods8090376

**Published:** 2019-09-01

**Authors:** Anh Dao Thi Phan, Mridusmita Chaliha, Yasmina Sultanbawa, Michael E. Netzel

**Affiliations:** ARC Training Centre for Uniquely Australian Foods, Queensland Alliance for Agriculture and Food Innovation, The University of Queensland, Health and Food Sciences Precinct, 39 Kessels Road, Coopers Plains, QLD 4108, Australia

**Keywords:** *Acca sellowiana*, feijoa fruit, proximate composition, polyphenols, vitamins, minerals, antimicrobial activity

## Abstract

The present study determined the chemical composition, bioactive compounds and biological properties of Australian grown feijoa (*Acca sellowiana*), including whole fruit with peel, fruit peel and pulp, in order to assess the nutritional quality and antimicrobial activity of this emerging subtropical fruit. Polyphenolic compounds and vitamins were determined by UHPLC-PDA-MS/MS, showing that the feijoa fruit not only contains high amounts of antioxidant flavonoids, but is also a valuable source of vitamin C (63 mg/100 g FW (fresh weight)) and pantothenic acid (0.2 mg/100 g FW). Feijoa fruit is also a good source of dietary fibre (6.8 g/100 g FW) and potassium (255 mg/100 g FW). The edible fruit peel possesses significantly (*p* < 0.05) higher amounts of antioxidant flavonoids and vitamin C than the fruit pulp. This is most likely the reason for the observed strong antimicrobial activity of the peel-extracts against a wide-range of food-spoilage microorganism. The consumption of feijoa fruit can deliver a considerable amount of bioactive compounds such as vitamin C, flavonoids and fibre, and therefore, may contribute to a healthy diet. Furthermore, the potential use of feijoa-peel as a natural food perseverative needs to be investigated in follow-up studies.

## 1. Introduction

Obesity, type 2 diabetes and cardiovascular disease are major chronic diseases in the developed world. Increased intake of fresh fruit and/or high-quality fruit products, resulting in increased consumption of bioactive compounds such as polyphenols, carotenoids, vitamins and dietary fibre, has been suggested as one approach to reduce the incidence of these conditions. The feijoa (*Acca sellowiana*) belongs to the family Myrtaceae and is commonly known as pineapple guava or guavasteen since it is related to the guava genus *Psidium guajava* L. [1]. The feijoa is native to South America around the highlands of the Uruguay and Brazilian border, but nowadays is widely distributed and cultivated in many countries, including Australia. The fruit of feijoa was described as a smooth and soft green skin fruit, with the juicy flesh being divided into a clear, gelatinous seed pulp and a firmer, slightly granular, opaque flesh nearer the skin [2]. However, the fruit has remained relatively unknown to many people around the world to this day.

Recent studies have reported that feijoa is a good source of vitamins (e.g., vitamin C), polyphenols, dietary fibre and essential minerals (e.g., potassium) [3]. For polyphenols, phenolic acids and flavonoids (e.g., flavone, catechin, quercetin-glycoside, procyanidin B1 and B2) have been found and identified as the major phenolic compounds in the feijoa fruits [4,5,6]. Interestingly, the bioactive components are not only present in the pulp, but also found at a relatively high level in the other biological tissues of the plant such as peel, leaf and flower bud [6,7,8,9]. Although the feijoa fruit peel is edible and can be a rich source of functional ingredients such as polyphenols and pectin fibre [4], it is considered as a by-product of food processing. Furthermore, feijoa is rich in characteristic aroma and volatile compounds such as methyl benzoate, ethyl butanoate and ethyl benzoate [10], giving this fruit an ‘unique’ flavour profile.

Apart from unique nutritional and sensory qualities, feijoa fruit also shows potential biological activity. Zhu [3] has published a comprehensive review that summarized the health-related properties of the feijoa plant both in vitro and in vivo. For example, feijoa fruit demonstrated antioxidant activity in male Sprague Dawley rats [11] and anti-inflammatory effects and superoxide anion generation in male Wistar rats [12]. Whilst information about the health benefits reported from in vivo studies is relatively limited, in vitro studies have shown a wide-range of biological properties such as anticancer, antidiabetic, antimicrobial, antioxidant and anti-inflammatory activities of the feijoa plant [5,6,13,14,15].

As feijoa fruit has become more popular and cultivated in Australia for fresh consumption and processing, a better understanding of its nutrient and phytochemical composition and subsequent potential bioactivity is crucial. Australia, compared to other countries and continents, has a ‘unique’ natural environment, which can have a significant impact on the nutritional quality and bioactivity of fruits and vegetables. Therefore, the aim of the present study was to determine the nutritional characteristics of Australian grown feijoa fruit, its antioxidant and antimicrobial properties, to generate important information for a better assessment of its nutritional ‘value’.

## 2. Materials and Methods

### 2.1. Materials

Ready-to-eat fresh feijoa fruits (approximately 5 kg; Supplementary Figure S1) were harvested randomly in Victoria (Australia) and provided by Produce Art Ltd. (Rocklea, QLD, Australia). Whole fruits, pulp and peel (after manual separation), were freeze-dried at −50 °C for 48 h (CSK Climatek, Darra, QLD, Australia) and ground to powder (Supplementary Figure S1). The powdered samples were stored at −35 °C until further analysis.

Commercial phenolic standards ((+/−)-catechin, ellagic acid, vanillic acid, p-coumaric acid, ferulic acid; ascorbic acid), α-tocopherol, sugar, and organic solvents (HPLC grade) were purchased from Sigma-Aldrich (Castle Hill, NSW, Australia).

Cultures of *Staphylococcus aureus* strain 6571 and *Escherichia coli* strain 9001 were obtained from the National Collection of Type Cultures (NCTC, Health Protection Agency Centre for Infection, London, UK), and *Candida albicans* (strain 90028) was sourced from the American Type Culture Collection (ATCC, In Vitro Technologies Pty Ltd., Noble Park, VIC, Australia).

### 2.2. Proximate Analysis 

The proximate composition of the whole fruit powder was analysed by Symbio Alliance Laboratories (Eight Mile Plains, QLD, Australia), a National Association of Testing Authorities (NATA) accredited laboratory that complies with the International Organization for Standardization/the International Electrotechnical Commission (ISO/IEC) 17025:2005. The analysis were carried out according to NATA approved in-house methods or appropriate Association of Official Analytical Chemists (AOAC) methods. Analysis of protein by AOAC method 990.03 [16]; fat by AOAC method 991.36 [17]; saturated, mono-unsaturated, poly-unsaturated and trans-fatty acids by gas chromatography with flame-ionization detection (in-house method CFH068.2); ash by AOAC method 923.03 [18]; minerals and heavy metals by inductively coupled plasma spectrometry (in-house methods ESI02 and ESM02, respectively); total dietary fibre by enzyme digestion and spectrophotometric in-house method (CF057); energy based on calculation from proximate data (in-house method CF030.1); crude fibre by AOAC method 962.09 [17]; dry matter using air-oven (in house method CF006.1); and selected B-vitamins by high performance liquid chromatography (in-house method CHF363).

### 2.3. Measurement of Physico-Chemical Parameters 

In order to measure the physico-chemical parameters, the fresh fruits (with peels) were blended into a puree using a Laboratory Blender Waring 8010S (Waring^®^ Laboratory Science, Torrington, CT, USA). The puree was used for the determination of total soluble solids (TSS) using a digital refractometer HI 96,804 HANNATM (Hanna Instruments Ltd., Leighton Buzzard, UK), pH and total acid content (TA; titrimetric method) using an automated titration system Metrohm 795 Karl Fischer Titrator System (Metrohm, Herisau, Switzerland).

### 2.4. Extraction and Analysis of Individual Polyphenols 

#### 2.4.1. Extraction of Free Phenolic Compounds 

Extraction of free phenolic compound was carried out as per the previously reported method [19], with a few minor modifications. Approximately 500 mg powdered samples were extracted with 80% methanol containing 1% HCl (*v*/*v*) on a reciprocating shaker (RP1812, Paton Scientific, Adelaide, SA, Australia) for 15 min in the dark at room temperature. Ultra-sonication was subsequently applied to the samples for 15 min, followed by centrifugation (3,900 rpm for 5 min; Eppendorf Centrifuge 5804, Hamburg, Germany). Supernatants were retained, whilst the residues were re-extracted twice with the procedure described above. The supernatants were combined and subjected to U(H)PLC-PDA-MS analysis and the total phenolic content (TPC) assay. The extraction was conducted in triplicate.

#### 2.4.2. Extraction of Bound Phenolic Compounds 

Bound phenolic compounds were extracted according to the method described by Adom and Liu [20] with slight modifications. Briefly, the residues obtained in 2.4.1. were subjected to alkaline hydrolysis for 1 h while shaking. After that, the samples were acidified to pH 2.0 (using concentrated HCl) and then ethyl acetate was added to further purify the released phenolic compounds. The ethyl acetate extracts were collected and dried under nitrogen at 40 °C in a dry block heater (DBH30D, Ratek Instruments Pty Ltd., Melbourne, VIC, Australia) and re-dissolved in 50% methanol containing 1% formic acid for further analysis.

#### 2.4.3. U(H)PLC-PDA-MS Analysis 

Analysis of individual (main) phenolic acids and flavonoids (free and bound) by UPLC-PDA followed the method of Gasperotti et al. [21], using a Waters Acquity^TM^ UPLC-PDA System (Waters, Milford, MA, USA). The compounds were separated on a Waters HSS-T3 column (100 × 2.1 mm i.d; 1.8 μm) maintained at 40 °C, with aqueous 0.1% formic acid (eluent A) and 0.1% formic acid in acetonitrile (eluent B). The gradient program (time (min), % B) was: (0.0, 5); (3.0, 5); (4.3, 20); (9.0, 45); (11.0, 0); (14.0, 0) with a flow rate of 0.4 mL/min.

Detected peaks in the feijoa samples were identified by a Thermo high resolution Q Exactive mass spectrometer equipped with a Dionex Ultimate 3000 UHPLC system (Thermo Fisher Scientific Australia Pty Ltd., Melbourne, VIC, Australia). A full scan in negative (ESI) ionization mode was acquired at a resolving power of 70,000 full width half maximum, followed by an MS2 scan range of *m*/*z* 100–1200 for the compounds of interest. The Thermo XcaliburTM software (Thermo Fisher Scientific) was used for data acquisition. The detected peeks were identified by matching spectrum, retention time, and MS data obtained from literature. External calibration curves were constructed from the polyphenolic standard solutions (0–2 mg/10 mL in methanol) for quantification, except for dihydroxyflavone, which was quantified as mg of catechin equivalent.

### 2.5. Total Phenolic Content (TPC) 

The TPC was determined in the ‘free’ and ‘bound’ extracts as previously reported [22,23], using a micro-plate absorbance reader (Sunrise Tecan, Maennedorf, Switzerland) at 700 nm. TPC was expressed as mg of gallic acid equivalents (GAE).

### 2.6. Analysis of Sugar

Sugar analysis was performed as previously reported [24,25], with a few minor modifications. Briefly, 1 g of powdered samples were incubated with hot water (60 °C) for 30 min in a sonication bath maintained at 60 °C, followed by centrifugation at 3900 rpm for 10 min. The supernatants were collected and subjected to Solid Phase Extraction (SPE; Bond Elut LRC-C18, 500 mg, Part No: 12113027, Agilent Technologies, Santa Clara, CA, USA). A Shimadzu HPLC Class VP system (Shimadzu Corp., Kyoto, Japan) coupled with a Shimadzu ELSD-LT detector was employed. ELSD parameters were set as follows: N_2_ low-flow nebulizer pressure 350 KPa, temperature 47 °C, gain 4. Sugar components were separated using a Luna C18-NH_2_ column (250 × 4.6 mm, 5 µm, Phenomenex, Lane Cove West, NSW, Australia) at 40 °C, with an isocratic elution (aqueous acetonitrile; 88%, *v*/*v*) at a flow rate of 2.5 mL/min.

### 2.7. Analysis of Vitamin E (Alpha-Tocopherol)

Extraction of α-tocopherol was performed according to the method described by Chun et al. [26] with slight modifications. Briefly, 0.5 g feijoa powder samples were extracted with ethanol containing 0.1% (*w*/*v*) butylated hydroxytoluene (BHT), followed by saponification using 30% KOH (*w*/*v*) in MeOH for 30 min in the dark at room temperature, while shaking at 100 rpm. NaCl 10% (*w*/*v*) was added to the tubes and the sample was extracted 4 times with a mixture of hexane/ethyl acetate (85:15, *v*/*v*) containing 0.1% BHT. The upper phase was collected and evaporated until dryness (under nitrogen stream). The dry extract was re-dissolved in ethanol containing 0.1% BHT prior to UHPLC-MS/MS analysis. A Shimadzu UHPLC system (Shimadzu Corp., Kyoto, Japan) equipped with a Shimadzu 8060 triple-stage quadrupole mass spectrometer was employed. The ESI source was operated in positive mode with multiple reaction monitoring (MRM) to identify and quantify α-tocopherol. Mass transition 429.3 → 165.2 (CE at −21 eV) was used for quantification. An isocratic flow of 0.1% formic acid in methanol at the flow rate of 0.17 mL/min was used to elute α-tocopherol through a Waters C18 BEH column (2.1 × 100 mm i.d, 1.7 µm; Waters, Milford, MA, USA) at 30 °C.

### 2.8. Analysis of Vitamin C (Ascorbic Acid)

Ascorbic acid (L-AA) extraction and analysis was conducted following the method published by Campos and co-workers [27], with slight modifications. Briefly, 200 mg feijoa powder sample was extracted with 3% meta-phosphoric acid containing 8% acetic acid and 1 mM ethylenediamine-tetraacetic acid (EDTA). The reduction of dehydroascorbic acid (DHAA), which was also present in the extracts/samples, to L-AA was performed following the method of Spinola et al. [28], prior to UPLC-PDA analysis. Total vitamin C (L-AA + DHAA) was determined using a Waters UPLC-PDA system and a Waters HSS-T3 column (100 × 2.1 mm i.d; 1.8 μm; 25 °C), with aqueous 0.1% formic acid as the mobile phase (0.3 mL/min) and isocratic elution. An external calibration curve of L-AA was used for quantification.

### 2.9. Antimicrobial Screening Test

Agar well diffusion assay was performed against the selected microorganisms. Isolated microbial colonies were grown on plate count agar plates (for *S. aureus* and *E. coli*) or potato dextrose agar (PDA) plates (for *C. albicans*) at appropriate growth temperatures (37 °C for bacteria and 30 °C for the fungi), for 16 h. The microbial growth was then diluted in sterile Phosphate Buffered Saline (PBS) and adjusted to an absorbance reading of 0.1 at 600 nm (corresponds to an inoculum of 10^4^ CFU/mL) using a spectrophotometer (Genesys 20, Thermo Fisher Scientific Australia Pty Ltd., Melbourne, VIC, Australia).

Freeze dried powders (approximately 1 g) of whole feijoa fruit, pulp or peel, were extracted twice with 10 mL water or methanol. Following the extraction, the water and methanolic extracts were evaporated at 60 °C and 40 °C, respectively in a miVac sample Duo concentrator (Genevac Ltd., Ipswich, UK) until dryness. Aqueous methanol solution 20% (*v*/*v*) was used to freshly reconstitute the dry extracts prior to antimicrobial test. 

For the antimicrobial assay, Mueller Hinton Agar (MHA) plates (Oxoid CM0337, Thermo Fisher Scientific, Melbourne, VIC, Australia) were impregnated with the adjusted microbial cultures aseptically. Wells of 11 mm diameter were made aseptically onto the inoculated plates. A volume of 100 µL of each of the extracts was added to the wells. The assay also included a mixture of penicillin and streptomycin (1 µg each) (Gibco, Life Technologies, Melbourne, VIC, Australia) and 10 µg amphotericin B (Sigma Aldrich Inc, Sydney, NSW, Australia) that were used as ‘antibiotic control ’ for bacteria and fungi, respectively. The aqueous methanol 20%, used for re-suspending, was also included to evaluate the effect of extracted solvent on the microbial growth. Plates were incubated overnight at the appropriate growth temperature.

At the end of the incubation period, the diameters of the inhibition zones formed around each well were determined and presented in mm. The zone of inhibition was categorized as low (1–6 mm), moderate (7–10 mm), high (11–15 mm), and very high antimicrobial activity (16–20 mm) [14].

### 2.10. Statistical Analysis

A one-way analysis of variance (ANOVA), using Minitab 17 for Windows (Minitab Pty Ltd., Sydney, NSW, Australia) was applied to test significant differences between the whole fruit, the pulp and the peel. Means were compared using Tukey’s least significant difference test at a 5% significance level.

## 3. Results and Discussion

### 3.1. Physico-Chemical Parameters

The physico-chemical parameters of the feijoa whole fruit puree are summarized in Table 1. The total acid (TA) content and total soluble solids (TSS) are important factors for fruit quality, whilst the ratio TSS:TA (or ripening index) is usually used for determination of the taste and palatability of the fruit and consequently the consumer acceptability. TSS and pH are in agreement with literature data [13,29,30]. However, the TA content was lower than the reported values of 12 feijoa cultivars grown in Italy [13], but was higher than that of the feijoa accessions grown in Brazil [29] and Colombia [30], probably reflecting differences in cultivars, growing conditions/environment, maturity and storage. The moisture content of the fresh feijoa is also in the same range as reported in the literature.

### 3.2. Proximate Analysis

Only the freeze-dried whole fruit powder was analyzed for total energy, protein, fat, minerals, dietary fibre, heavy metals, dry matter and ash content. The proximate results of the present study are similar to that reported in the literature, as shown in Table 2.

The results of proximate analysis showed that feijoa is a good source of dietary fibre with 34.6 g/100 g DW (Table 2), being equivalent to 6.8 g/100 g FW (calculated based on the moisture content given in Table 1). According to Food Standards Australia New Zealand, if a serving of the food contains at least 4 g of dietary fibre, it can be considered as a good source of dietary fibre. Based on this, feijoa is definitely a valuable fruit for a healthy diet. The high dietary fibre content of the feijoa whole fruit powder might be mainly derived from the peel. The adequate intake (AI) for dietary fibre in Australia and New Zealand is 25–30 g/day for adults [31], which means that a serving size of 250 g feijoa (whole) fruit can deliver 50% of the AI for adults. It is well documented that an adequate intake of dietary fibre is essential for a healthy gut and has also been related to a reduced risk for developing common ‘life-style diseases’ such as heart disease, certain cancers and type 2 diabetes. However, the protein (0.73 g/100 g FW) and fat (0.43 g/100 g FW) content of this powder sample are relatively low. Interestingly, the proximate data of the Australian grown feijoa fruits (Table 2) are similar to that in the USDA Food Composition Database reported by Zhu [3].

### 3.3. Minerals and Heavy Metals

The analyzed minerals and heavy metals are summarized in Table 3. Potassium was found to be highest among the seven minerals tested, followed by calcium, magnesium, sodium, iron, zinc and iodine. Furthermore, the potassium content in the Australian grown feijoa fruit was higher than that reported by Zhu [3] (255 mg/100 g FW versus 172 mg/100 g FW). Relevant (nutrition) information in regard to AI, RDA, EAR and UL are also provided in Table 3. Aluminium was found to be highest (0.25 mg/kg FW) among the six heavy metals tested followed by lead, arsenic and chromium (both <0.005 mg), mercury and cadmium (both <0.002 mg). As shown in Table 3, the levels of heavy metals found in the Australian grown feijoa fruits are considerably lower than the reported ULs.

### 3.4. Sugar Components

Sugar is not only important for the ‘pure’ sweetness of fruits, but also for its flavor and sensory attributes and subsequent consumer acceptance. Therefore, a detailed sugar analysis is necessary to better understand the relationship between the individual sugar profile in a fruit and its impact on aroma and taste. Individual sugar components and the total sugar content are summarized in Table 4, showing that sucrose is the main sugar in feijoa whole fruit and pulp with up to 50% of the total sugar content. Previously, Oksana et al. [36] reported a similar sugar profile and sugar concentrations in 18 different feijoa fruits that varied in ripening stage and fruit mass (Table 4). Unlike other common fruits such as grapes or guava, in which glucose and fructose are major sugars [37,38], feijoa fruit is similar to strawberry with sucrose as the main sugar component [24]. The contents of sucrose and total sugar in the peel were significantly (*p* < 0.05) lower than in the whole fruit and pulp (Table 4).

### 3.5. Total Phenolic Content (TPC)

**Figure 1:**
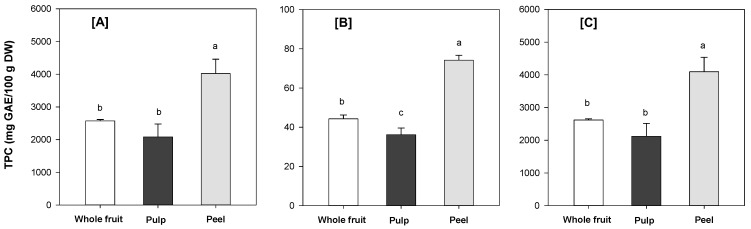
Total phenolic content (TPC): (**A**) Free, (**B**) Bound and (**C**) Total (free + bound) in powdered feijoa whole fruit, pulp and peel. The TPC was calculated on dry weight basis. Data are means ± SD (*n* = 3). Different letters in the same figure indicate significant differences at α = 0.05.

The TPC results (free, bound and total) are summarized in Figure 1. After conversion to fresh weight, the TPC in Australian grown feijoa fruit was higher than that reported in several previous studies: 515 mg GAE/100 g FW (present study) versus. 93–251 mg GAE/100 g FW [13] and 197–359 mg GAE/100 g FW [39], but relatively similar to the reported TPC for flesh, peel and whole fruit of New Zealand grown feijoa cultivars [6]. Again, different cultivars/genotypes, growing conditions/environment, locations, maturity as well as pre-and post-harvest treatment of the fruits are most likely the reasons for the observed difference in TPC.

Interestingly, the free-TPC was considerably higher than the bound-TPC in the whole fruit, pulp and peel. This may affect the bioaccessibility (matrix-release) and subsequently, the bioavailability of feijoa fruit polyphenols (potentially more bioaccessible and better bioavailable). However, this needs to be substantiated in follow-up studies using in vitro digestion models and human clinical trials. The feijoa peel had the highest TPC (*p* < 0.05), indicating a high content of bioactive (poly)phenols which is in agreement with previous finding [6]. The potential utilization of feijoa peel as a source of functional ingredients for food and nutraceutical applications should be investigated further.

### 3.6. Individual Phenolic Compounds

**Figure 2:**
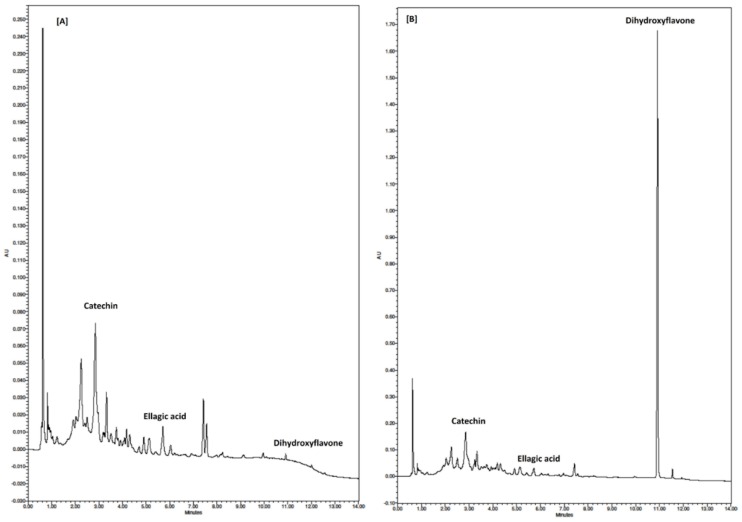
Representative UHPLC-PDA chromatograms of (**A**) feijoa pulp and (**B**) feijoa peel showing the (main) phenolic compounds detected in the respective extracts.

**Figure 3:**
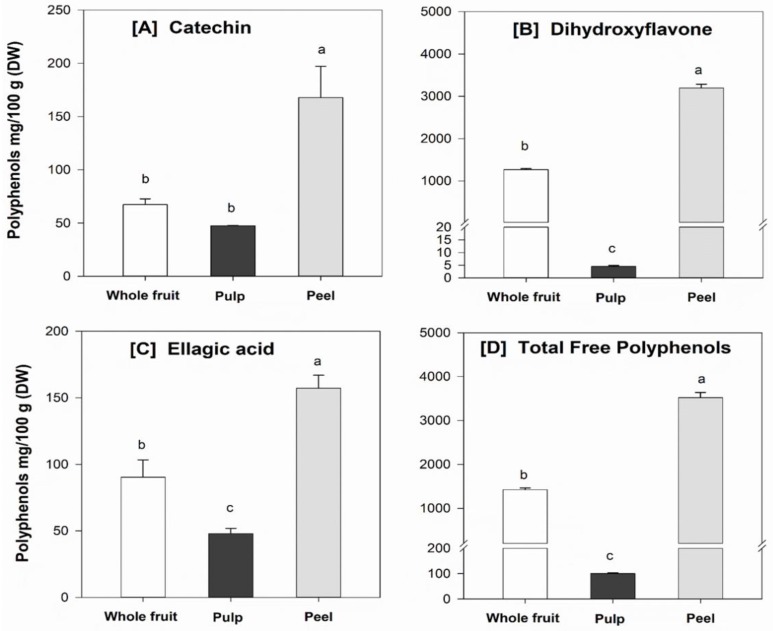
Free (main) phenolic compounds, including (**A**) catechin, (**B**) dihydroxyflavone, (**C**) ellagic acid, and (**D**) total amount of free polyphenols in the powdered feijoa fruit samples. Data are means ± SD, *n* = 3. Different letters in the same figure indicate significant differences at α = 0.05.

**Figure 4:**
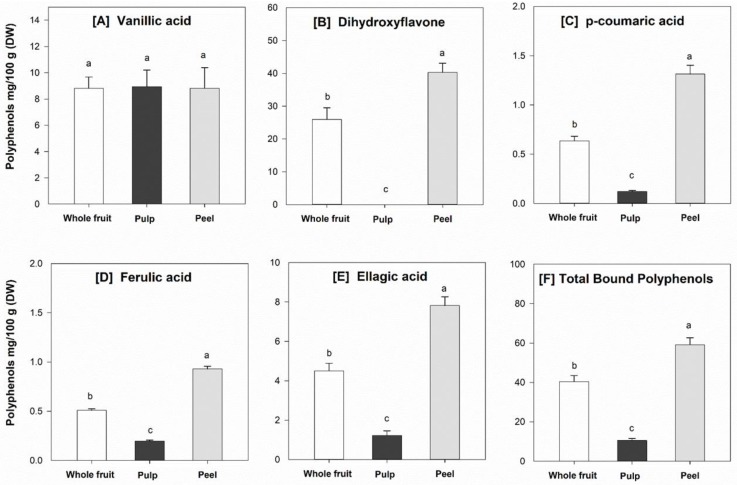
Bound (main) phenolic compounds, including (**A**) vanillic acid, (**B**) dihydroxyflavone, (**C**) p-coumaric acid, (**D**) ferulic acid, (**E**) ellagic acid, and (**F**) total amount of bound polyphenols in the powdered feijoa fruit samples. Data are means ± SD, *n* = 3. Different letters in the same figure indicate significant differences at α = 0.05.

Results from chromatographic and mass spectrometric analysis show that dihydroxyflavone (*m*/*z* 253), catechin (*m*/*z* 289) and ellagic acid (*m*/*z* 301) could be identified as the main free phenolic compounds in the powdered feijoa fruit samples (Figure 2), whereas dihydroxyflavone, vanillic acid (*m*/*z* 167), p-coumaric acid (*m*/*z* 163), ferulic acid (*m*/*z* 193) and ellagic acid were the main bound polyphenolics. Interestingly, dihydroxyflavone (free and bound) was found predominantly in the peel of the feijoa fruit, with up to 90% of the total amount (Figure 3 and Figure 4). Consistent with the TPC results, the concentrations of individual phenolics were significantly (*p* < 0.05) higher in the peel compared to the fruit pulp. These findings are in agreement with recent studies that have also identified flavone, catechin and ellagic acid as the major phenolic compounds in feijoa fruit [5,6,40]. It has been reported that 7,8-dihydroxyflavone exerts strong neuroprotective effects in monkeys [41], is effective in early brain trauma recovery in male rats [42], and has potential anticancer activity [40]. However, further studies on the potential health benefits of feijoa fruit in humans and the exact mode of action of its bioactive compounds are warranted.

### 3.7. Vitamins

The results of the vitamin analysis are summarized in Table 5 and Table 6. B-vitamins are crucial in many metabolic and physiological processes and can act as coenzymes in the energy metabolism (vitamin B1, B2, B3, B5 and B7), production of new cells (vitamin B6 and B12), protein metabolism (vitamin B6), and are essential for a functioning nervous system (vitamin B1, B3 and B12) [43]. Pantothenic acid (vitamin B5) was highest among the seven B-vitamins tested and a 250 g serve of feijoa fruit would deliver almost 14% of the RDI for adults (Table 5).

The vitamin C content of feijoa fruit was considerably higher than previously reported data (Table 6). According to the obtained results, 100 g fresh feijoa fruit (containing the fruit peel) would supply 140% (62.8 mg) of the RDI of vitamin C for adults (45 mg/day [31]). Besides vitamin C, feijoa also contained vitamin E (α-tocopherol). It has been shown that a high intake of vitamin E is correlated with a reduced risk to develop non-communicable diseases [44]. Alpha-tocopherol, the most biologically active form of vitamin E, was found as the main tocopherol constituent in the feijoa fruit samples. Other tocopherol-forms were also present in the feijoa samples (data not shown), however, in very low concentrations (at or below the limit of quantification) and therefore not quantified. To the best of our knowledge, there is no previous investigation on the vitamin E content in feijoa pulp and peel. The available data from the USDA (Table 6) do not provide clear information whether the analyzed fruit contained the peel or not. Previously, the presence of α-tocopherol (qualitative analysis only) in lipid extracts of feijoa leaves has been reported [45].

Based on the present results, Australian grown feijoa fruit can be considered as an ‘excellent’ source of vitamin C, but is not a major source of vitamin E like tomatoes (containing up to 8 mg vitamin E/100 g FW [46]). The RDI for vitamin E for adults is 7–10 mg/day [31]. Furthermore, the peel sample had significantly (*p* < 0.05) higher vitamin C and vitamin E levels than the feijoa pulp. This ‘trend’ was similar to that already observed in the TPC/polyphenol-results.

### 3.8. Antimicrobial Activity

Antimicrobial efficacy of water and methanolic extracts of feijoa whole fruit, pulp and peel were determined against three microorganisms: A Gram-positive *S. aureus*, a Gram-negative *E coli* and a fungi *C. albicans* (Supplementary Figure S2). The inhibition zones varied from 11.9 to 23.4 mm (Table 7), suggesting a ‘high’ to ‘very high’ antimicrobial activity of feijoa-extracts against the three microorganisms tested. However, the water extracts of feijoa failed to show any activity against *E. coli* and *C. albicans*. Overall, the methanolic extracts of feijoa peel had the strongest antimicrobial activity of all samples/extracts, followed by the methanolic extracts of feijoa whole fruit.

*S. aureus* is an important pathogen responsible for causing foodborne diseases in humans. *S. aureus* produces enterotoxins leading to food poisoning in humans [47]. Typically, humans are asymptomatic carriers of enterotoxigenic *S. aureus* and carry it in nose, throat, and skin. Therefore, food handlers can be an important source of food contamination. *E. coli* is an important food related pathogen. The genus *Candida* comprises of ~200 species of fungi with distinguished morphological, biochemical and genetic characteristics. They are known to be opportunistic pathogens, affecting mainly immunocompromised individuals [48,49]. This study included *C. albicans* as reference fungi to assess the efficacy of feijoa extracts against fungi.

Antimicrobial assessment indicated that methanolic extracts of feijoa tissues have broad antimicrobial efficacy. The strong antimicrobial efficacy of the methanolic feijoa peel-extract is in agreement with Motohashi et al., [9], who also reported a significant inhibitory effect of methanolic extracts from feijoa peel against *S. aureus*, *E. coli* and *C. albicans*. Future studies are warranted to identify individual bioactive compounds, released in different extracted solvents, that might contribute to observed antimicrobial activity.

The results also indicate that the Gram-positive *S. aureus* was more susceptible to the tested extracts compared to the Gram-negative *E. coli.* The difference in susceptibility between Gram-positive and Gram-negative to feijoa extracts, can be attributed to the difference in the bacterial morphology. Gram-negative bacteria like *E. coli*, contains an outer phospholipid membrane, which can act as an effective barrier against hydrophobic molecules from penetrating the cell wall [50]. This complex outer layer of Gram-negative bacteria allows them to be more resistant to plant extracts and essential oils with antimicrobial activity 

The antimicrobial efficacy of the feijoa samples could be attributed to the presence of bioactive phytochemicals. As mentioned before, feijoa peel showed the strongest antimicrobial efficacy among the tested samples, which correlates well with our observation that the feijoa peel in its powdered or fresh form possesses the highest polyphenol and vitamin C concentration.

## 4. Conclusions

Our findings suggest that Australian grown feijoa fruits are a valuable source of dietary fibre, minerals (potassium), polyphenols (e.g., flavones), vitamin C and B5, and exhibit a broad spectrum of antimicrobial activity. The fruit peels have potential to be utilized for the extraction of functional ingredients for the food and nutraceutical industries. The observed broad spectrum of antimicrobial activity of feijoa fruit extracts is promising with regard to the potential use as natural food preservatives. The assessment of the digestive stability, bioaccessibility (matrix release), bioavailability and subsequent bioactivity both in vitro and in vivo are strongly recommended to get a better understanding of the nutritional value of feijoa, an emerging fruit in the Australian fresh fruit market.

## Figures and Tables

**Table 1:** Physico-chemical parameters of fresh feijoa whole fruit puree.

Parameters	Fresh Whole Fruit Puree *	Literature Data
TSS (%)	13.9 ± 0.1	10.08–12.89 [13]
		9.3–12.5 [29]
		11.19–13.35 [30]
pH	3.1 ± 0.03	2.45–3.68 [7]
		3.2–3.4 [29]
TA (g citric acid Eq/100 g)	2.0 ± 0.05	4.05–6.7 [13]
		0.9–1.5 [29]
		1.58–1.93 [30]
TSS: TA	7.2 ± 0.2	1.9–3.35 [13]
		8.5–12.1 [29]
Moisture content (%)	80.3 ± 0.8	83.3 [3]

Eq: equivalent; (*) Data are means ± SD (*n* = 3).

**Table 2:** Proximate of the feijoa whole fruit powder.

Proximate Composition		Quantity per 100 g DW	Quantity per 100 g FW *	Literature Data [3] per 100 g FW
Energy		1203 kJ	237 kJ	n/a
		288 Cal	57 Cal	61 Cal
Protein		3.7 g	0.73 g	0.71 g
Fat	Total fat content	2.2 g	0.43 g	0.42 g
	Saturated fatty acids	0.6 g	0.12 g	0.104 g
	Monounsaturated fatty acids	0.3 g	0.058 g	0.056 g
	Polyunsaturated fatty acids	1.3 g	0.26 g	0.136 g
	Trans fatty acids	<0.1 g	<0.02 g	0 g
Dietary fibre	Total dietary fibre Crude fibre	34.6 g 20.4 g	6.8 g 4.01 g	6.4 g n/a
Ash		3.5%	0.01 g	n/a

Data area means of duplicate analysis; DW: Dry weight; FW: Fresh weight; (*) Results in DW converted to FW based on the moisture content given in Table 1; (n/a): Not available.

**Table 3:** Minerals and heavy metals in the powdered feijoa whole fruit sample.

Minerals and Heavy Metals		Quantity per kg DW	Quantity per kg FW *	Relative Percentage per 100 g FW ≠	Nutrition Information **
Minerals	Sodium (Na)	96 mg	18.87 mg	0.15	1.3 g/day AI [32]
	Potassium (K)	13,000 mg	2,556 mg	5.4	4.7 g/day AI [32]
	Iron (Fe)	12.8 mg	2.5 mg	3.1	8 mg/day RDA [32]
	Calcium (Ca)	940 mg	185 mg	1.5	1200 mg/day AI [32]
	Magnesium (Mg)	614 mg	121 mg	3.5	350 mg/day EAR [32]
	Zinc (Zn)	4.3 mg	0.9 mg	0.82	11 mg/day RDA [32]
	Iodine (I)	0.4 mg	0.08 mg	8.4	95 µg/day EAR [32]
Heavy metals	Mercury (Hg)	<0.01 mg	<0.002 mg		5 µg/kg BW/week UL [33]
	Lead (Pb)	0.11 mg	0.022 mg		25 µg/kg BW/week UL [33]
	Cadmium (Cd)	<0.01 mg	<0.002 mg		2.5 µg/kg BW/week UL [34]
	Arsenic (As)	<0.025 mg	<0.005 mg		-
	Aluminium (Al)	1.29 mg	0.25 mg		1.0 mg/kg BW/week UL [35]
	Chromium (Cr)	<0.025 mg	<0.005 mg		25-35 µg/kg day AI [32]

Data area means of duplicate analysis; DW: Dry weight; FW: Fresh weight; ≠ Relative percentage in relation to the nutrition information given in the adjacent column; * Results in DW converted to FW based on the moisture content given in Table 1; ** RDA: Recommended Dietary Allowance; AI: Adequate Intake; UL: Tolerable Upper Intake Level; EAR: Estimated Average Requirement; BW: Body weight; (-): Not available.

**Table 4:** Sugar content in feijoa fruit (g/100 g).

Sugar Components	Whole Fruit	Pulp	Peel	Literature Data (Whole Fresh Fruit)
Fructose	11.9 ± 0.4 a *	12.3 ± 0.6 a	12.2 ± 0.1 a	
	(2.3) **	(2.3)	(2.3)	1.4–4.3 g/100 g FW [36]
Glucose	13.4 ± 0.5 a	13.7 ± 0.4 a	13.2 ± 0.3 a	
	(2.6)	(2.7)	(2.6)	0.07–1.5 g/100 g FW [36]
Sucrose	25.9 ± 1.0 b	29.0 ± 0.9 a	11.5 ± 0.3 c	
	(5.1)	(5.7)	(2.3)	2.15–5.9 g/100 g FW [36]
Total sugars	51.2 ± 1.3 b	55.0 ± 1.6 a	36.9 ± 1.5 c	
	(10.1)	(10.8)	(7.3)	

Data are means ± SD (*n* = 3); Calculated based on * DW: Dry weight and ** FW: Fresh weight; Different letters at the same row indicate significant differences at α = 0.05.

**Table 5:** Selected B-vitamins in feijoa whole fruit powder.

Vitamins	Quantity (per 100 g) *		Nutrition Information [31] (RDI for Adults)
	Whole Fruit (DW)	Whole Fruit (FW)	
B1 (Thiamin)	<5.0 µg	<1 µg	1.1–1.2 mg/day
B2 (Riboflavin)	<5.0 µg	<1 µg	1.6 mg/day
B3 (Niacin)	270 µg	53.1 µg	14–16 mg/day
B5 (Pantothenic acid)	1100 µg	216.3 µg	4–6 mg/day
B6 (Pyridoxine)	190 µg	37.4 µg	1.7 mg/day
B7 (Biotin)	<5.0 µg	<1 µg	25–30 µg/day
B12 (Cyanocobalamin)	<5.0 µg	<1 µg	2.4 µg/day

Data are means of duplicate analysis; * Results in DW converted to FW based on the moisture content given in Table 1; RDI: Recommend Dietary Intake.

**Table 6:** Vitamin C and E in the powdered feijoa fruit samples.

Sample	Vitamin C (L-AA + DHAA)		Literature Data (mg/100 g FW)	Vitamin E (α-Tocopherol)		Literature Data (mg/100 g FW)
	(mg/100 g DW) *	(mg/100 g FW) *		(mg/100 g DW) *	(mg/100 g FW) *	
Whole fruit powder	319.2 ± 2.5 b	62.8 ± 0.4 b	32.9 [43]	1.41 ± 0.11 b	0.28 ± 0.02 b	0.16 [43]
			27.9–39.9 [7]			
Pulp powder	281.1 ± 0.6 c	51.8 ± 0.1 c	38.7–92.5 [13]	0.27 ± 0.03 c	0.05 ± 0.01 c	n/a
Peel powder	469.4 ± 4.3 a	95 ± 0.6 a	63.5–101 [13]	2.27 ± 0.14 a	0.45 ± 0.03 a	n/a

* Data are means ± SD (*n* = 3); DW: Dry weight; FW: Fresh weight; Results in DW converted to FW based on the moisture content given in Table 1; Different letters at the same column indicate significant differences at α = 0.05; n/a: Not available.

**Table 7:** Inhibition zones of the methanolic and water extracts from different tissues of feijoa fruit.

Samples	*E. coli*		*S. aureus*		*C. albicans*	
	MeOH	Water	MeOH	Water	MeOH	Water
Whole fruit powder	11.9 ± 0.2 b *	-	23.1 ± 0.8 b	20.1 ± 0.1 b	15.5 ± 1.2 a	-
Pulp powder	-	-	22.7 ± 0.3 b	18.9 ± 0.2 c	-	-
Peel powder	14.7 ± 1.1 a	-	26.5 ± 0.2 a	23.4 ± 0 a	15.6 ± 3.2 a	-
Antibiotic control	29.2		55.8		27.1	
Methanol (20%, *v*/*v*)	-		-		-	

Data are means ± SD, *n* = 3; MeOH: Methanolic extracts; (*) Different letters in the same column indicate significant difference at α = 0.05; (-) No zone of inhibition was observed. The zone of inhibition was categorized as low (1–6 mm), moderate (7–10 mm), high (11–15 mm), and very high antimicrobial activity (16–20 mm).

## Supplementary Materials

The following are available online at https://www.mdpi.com/2304-8158/8/9/376/s1, Figure S1: Feijoa samples: fresh fruit and freeze-dried fruit powder, Figure S2: representative photos showing the inhibitory activity of feijoa extracts (water and methanol) against gram-positive and gram-negative bacteria and yeast. Samples tested included (2.1)–Whole fruit powder, (2.2)–Pulp powder, (2.3)–Skin powder, and (2.4)–Fresh whole fruit puree.
