# Supplementary material for: Nutritional Characteristics and Antimicrobial Activity of Australian Grown Feijoa (Acca sellowiana)
Source: Foods. 2019 Sep 1;8(9):376. doi: 10.3390/foods8090376 (PMC6770449; doi:10.3390/foods8090376)
Supplement: Supplementary file 1 [file foods-08-00376-s001.pdf]

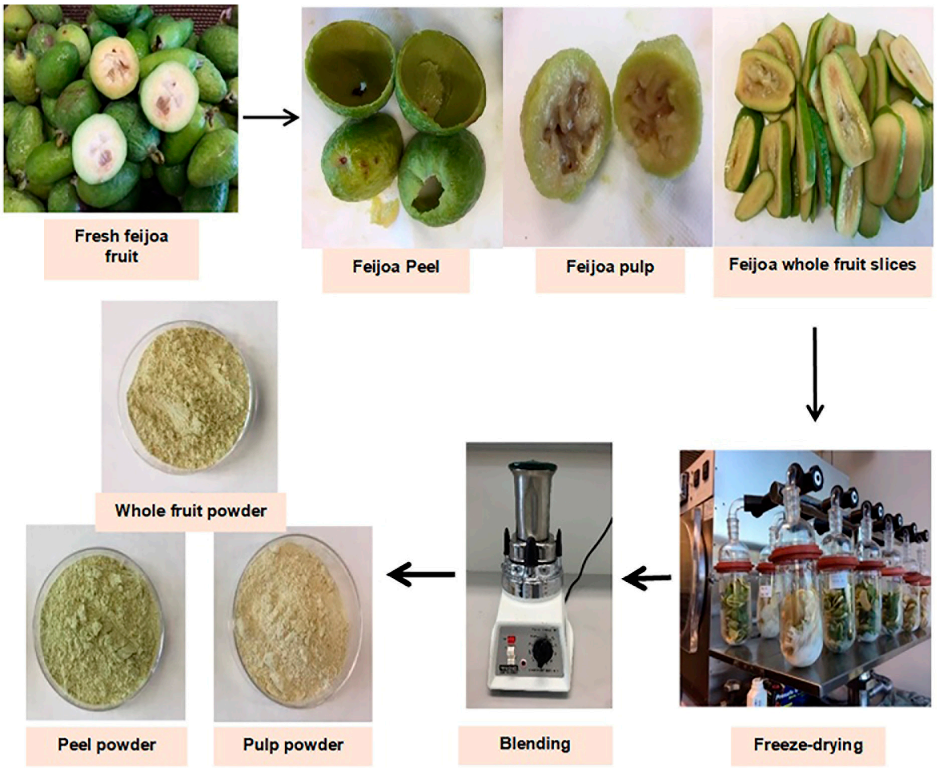

Supplementary Figure 1: Feijoa samples: fresh fruit and freeze-dried fruit powder

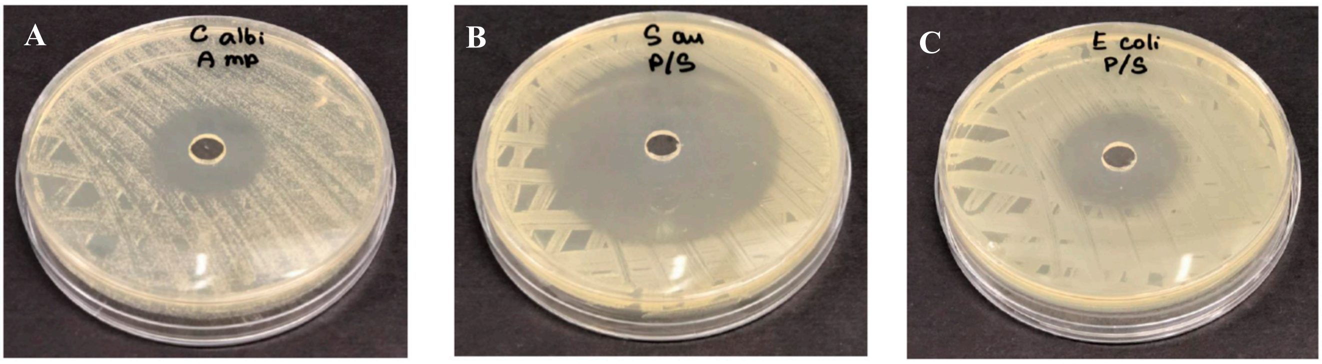

(A, B, C) inhibition of antibiotic controls against *C. albicans*, *S. aureus* and *E. coli*, respectively

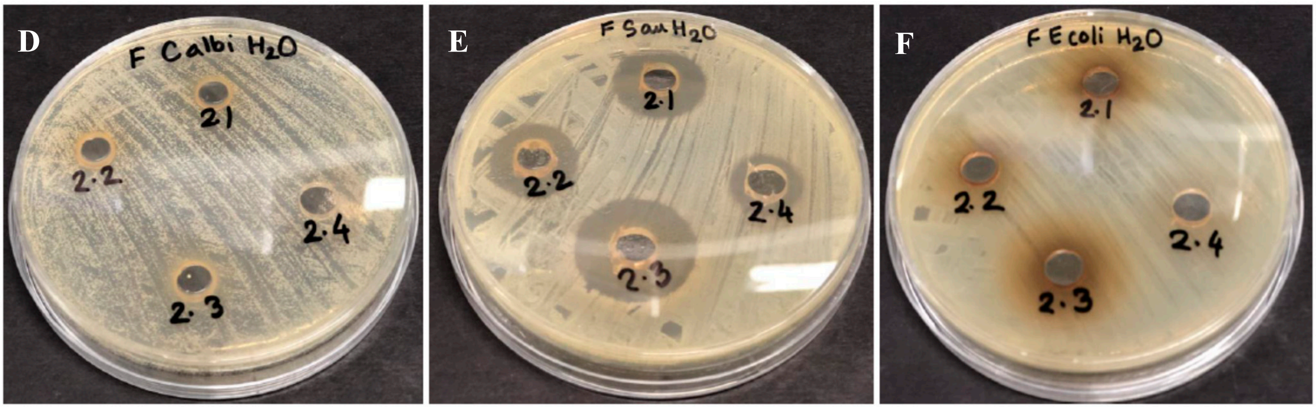

(D, E, F) Inhibition of Feijoa-water extracts against *C. albicans, S. aureus and E. coli*, respectively

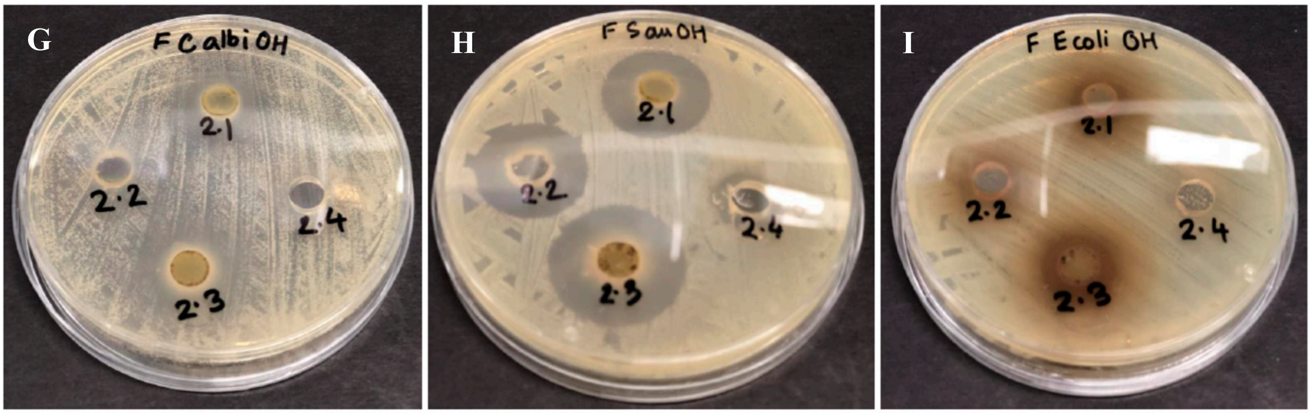

(G, H, I) Inhibition of Feijoa-methanolic extracts against *C. albicans, S. aureus and E. coli*, respectively

Supplementary Figure 2: representative photos showing the inhibitory activity of feijoa extracts (water and methanol) against gram-positive and gram-negative bacteria and yeast. Samples tested included (2.1)–Whole fruit powder, (2.2)–Pulp powder, (2.3)–Skin powder, and (2.4)–Fresh whole fruit puree.
